# The Level of Knowledge and Attitude Regarding Family Planning Methods Among Married Men and Women in the United Arab Emirates

**DOI:** 10.7759/cureus.105232

**Published:** 2026-03-14

**Authors:** Sarah Shahzad, Mohamed Ashraf, Mohamed Elkhouly, Abdulrahman Abusalem, Shatha AlSharbatti

**Affiliations:** 1 College of Medicine, Gulf Medical University, Ajman, ARE; 2 Department of Community Medicine, Gulf Medical University, Ajman, ARE

**Keywords:** contraceptive methods, family planning, married, men, women

## Abstract

Introduction

There is a lack of sufficient data regarding the knowledge levels and attitudes of married couples towards family planning (FP) in the United Arab Emirates (UAE). Researchers have highlighted an unmet demand for contraception and have demonstrated that married couples who need FP services have not adopted them due to insufficient knowledge. This study aimed to evaluate the knowledge levels and attitudes related to FP among married adults attending Thumbay Healthcare facilities in the UAE, and to examine the sociodemographic factors associated with them.

Methods

A cross-sectional study design was employed and included married men and women aged 18 years and above who provided informed consent. Institutional Review Board (IRB) approval was obtained before enrolling participants (IRB/COM/STD/65/April-2022). A validated and pilot-tested structured questionnaire was used. Chi-square test analysis was performed.

Results

We surveyed 487 participants, with the majority aged 31 to 50 years (n = 304, 62.4%), holding a university-level education or higher (n = 427, 87.7%), employed in non-medical sectors (n = 382, 78.4%), and earning a monthly family income of 15,000 to 25,000 AED (n = 156, 32%). The knowledge level of 253 participants (51%) was categorized as low or below average. Significant associations were found between knowledge and age, education level, nationality, occupation, duration of marriage, number of children, family income, and household type. Over half of the participants demonstrated a positive attitude (n = 268, 55%) and expressed interest in learning more about FP. Factors associated with a positive attitude were being male, Southeast Asian nationality, having a university-level education or higher, being employed, a marriage duration of five to nine years, having no children, and living in a nuclear family household. The internet and media (n = 286, 58.7%) were identified as the primary sources of information. Hesitancy or refusal to accept FP methods was primarily attributed to family or spousal taboos surrounding the topic (n = 349, 71%) and misinformation (n = 299, 61.4%).

Conclusions

Although the knowledge level among most participants was low or below average (n = 253, 51%), over half of the participants demonstrated a positive attitude (n = 268, 55%) and expressed interest in learning more about FP. Sociodemographic, economic, and family characteristics were associated with both knowledge and attitude. The findings highlight opportunities for implementing awareness campaigns and educational programs to improve understanding of the topic. Further research examining contraceptive practices and related behavioral outcomes would help determine the effectiveness of such initiatives.

## Introduction

The World Health Organization (WHO) has indicated that among the 1.9 billion women of reproductive age worldwide in 2019, 1.1 billion required family planning (FP). Of these, 842 million (76%) were using contraceptive methods, while 270 million (24%) had an unmet need for contraception. The study also revealed that many married couples who require FP have not adopted it due to insufficient knowledge [1]. Authors in Tanzania found that sociodemographic factors were associated with FP knowledge among women of childbearing age [2]. 

A study conducted in northern Nigeria revealed that knowledge of contraceptive methods among respondents was generally high, with 63.6% reporting awareness of at least one method. Knowledge of FP methods was associated with place of residence and education [3]. These findings were similar to those reported in another study in the literature [4], where formal education was linked to greater knowledge of contraceptive methods. A total of 1064 (68%) respondents reported knowing how to use contraceptives, with more women (77%) demonstrating knowledge compared with men [4].

Further studies show that the majority of married women demonstrate a positive attitude towards FP [5-7]. In one study, the reported reasons for rejecting FP methods included fear of side effects (22.2%), desire for more children (14%), health conditions (9.5%), financial problems (8%), and husband disapproval (5%) [5]. It was noted that women’s attitudes toward FP are influenced by education and previous pregnancy experiences. Fear of side effects and spousal opposition were identified as important factors highlighted by many women [6]. This contrasts with findings in other literature, where spousal approval was reported to be very high [8]. A study from Quetta, Pakistan, among married women demonstrated that the illiteracy level was 41.2% and impacted women’s knowledge and attitude; however, 98.8% of participants agreed that FP is beneficial. Similar to previously mentioned studies, the husband was noted as being the dominant factor in approving family size and FP methods [9]. 

A study from Abu Dhabi, United Arab Emirates (UAE) [10] demonstrated lower attitude levels toward FP than the aforementioned studies from Saudi Arabia. Notably, 92% of women believed that birth control education should be provided to all women before marriage. However, 46.1% of women believed that contraceptive methods may have negative health effects and therefore should not be used. An important factor identified was that women who had never used contraception showed a significantly higher proportion of negative attitudes. Despite the lower attitude levels, it was revealed that nearly 50% of participants reported using contraception, which is higher than in some other studies and comparable to findings reported in studies from Saudi Arabia [7,8]. 

The results obtained in this study may be beneficial for researchers and healthcare providers in planning health promotion programs that address gaps in knowledge and correct misinformation, thereby improving reproductive health awareness in the United Arab Emirates. Therefore, this study aimed to evaluate the level of knowledge regarding FP methods among married men and women attending Thumbay Healthcare facilities in the UAE; to assess attitudes toward FP methods; to identify the sociodemographic factors associated with knowledge and attitudes; and to examine the relationship between knowledge and attitudes toward FP methods.

## Materials and methods

Study design and setting

Our study used a cross-sectional design to assess the levels of knowledge and attitudes, as well as associated factors, among married men and women attending Thumbay hospitals, clinics, and centers in the UAE. A convenience sampling strategy was used to recruit participants. Eligible individuals attending outpatient clinics at Thumbay facilities were approached in the waiting areas and invited to participate in the study. Those who agreed were given a self-administered questionnaire. Participation was voluntary, and informed consent was obtained before completing the survey. Institutional Review Board approval was secured before the enrollment of participants (IRB/COM/STD/65/April-2022). Data collection was carried out over approximately 10 months, between April 15, 2022, and February 25, 2023. 

Study population

The inclusion criteria comprised married men and women of any nationality, aged 18 years or older, attending Thumbay hospitals, clinics, and centers, who agreed to provide informed consent. The exclusion criteria included unmarried adults, individuals who declined to provide informed consent, and those who submitted incomplete questionnaires.

Sample size

A minimum sample size of 482 participants was calculated based on a 5% margin of error and an assumed prevalence of 0.24%, using the formula n = Z²pq / L², where n represents the required sample size, P represents the estimated prevalence, q = 1 - P, L² represents the absolute margin of error, and Z corresponds to a 95% confidence level (1.96). After accounting for a potential 10% refusal rate, the final minimum sample size was determined to be 482 participants. A total of 487 participants completed the questionnaire. The response rate was not formally documented.

Study instrument

Self-administered questionnaires were developed by the research team and included three main components: sociodemography, knowledge, and attitudes toward family planning methods (Appendix 1). The first section collected sociodemographic information, including family characteristics. In the second section, participants were asked to rate their perceived knowledge across six domains of family planning (items 11-16): goal of FP, effectiveness of different FP methods, side effects of different FP methods, how to use different FP methods, precautions to be considered on using some FP methods, and the effect of FP methods on fertility and risk of sexually transmitted diseases/genital infection.

Participants rated their knowledge of the six domains using a 6-point Likert scale, ranging from very high (6) to never heard of (1). The scores were divided into four quartiles, with the lowest two quartiles categorized as “low” and the highest two as “high”. These categories were used in the analysis. Participants were also asked to indicate their sources of knowledge about family planning (item 17) and whether they discussed family planning with their spouses (item 18). The third section included 15 statements (items 19-33) to assess participants’ attitudes, using a 5-point Likert scale ranging from strongly agree (5) to strongly disagree (1). Scores above the median were classified as “positive attitude”, while scores equal to or below the median were classified as “negative attitude”. Participants were also asked to provide their opinions on reasons why people might rarely discuss or refuse family planning and contraceptive methods, with multiple-selection options (item 34).

The questionnaire was validated by one family physician and two obstetric and gynecologic physicians. The validated questionnaire was pilot-tested with five married men and women to assess the clarity of the questions and the time required to complete it. Minor adjustments were made based on the feedback. The questionnaire was made available in both an online digital format and a printed version. The full questionnaire is provided in Appendix 1. 

Analytical approach

Questionnaires with incomplete responses were excluded from the final analysis. Data were entered into a Microsoft Excel spreadsheet and analyzed using IBM SPSS for Windows, version 29 (IBM Corp., Armonk, NY). Descriptive and inferential statistics were applied. The Chi-square (χ²) test was used to assess associations between variables. Data were presented using charts, graphs, and tables. A p-value less than 0.05 was considered statistically significant.

## Results

Sociodemographic characteristics of participants

This study enrolled a total of 487 participants. Most of the participants were between 31 and 50 years old (62.4%), had completed a university degree or higher education (87.7%), were employed in non-medical fields (78.4%), and originated from Southeast Asia (60.0%). Regarding gender, the study sample included 263 male participants (54.0%) and 224 female participants (46.0%). Concerning family income, the largest group (32.0%) reported a monthly income between AED 15,000 and 25,000.

Distribution of family characteristics 

Regarding the duration of marriage, most participants (61.2%) had been married for fewer than five years. Over half of the participants (54.8%) had one or two children, while 26.1% had no children. Analysis of birth spacing showed that the majority of participants (79.7%) had one to two years between children. Data on family structure indicated that 80.5% of participants resided in nuclear families, whereas 19.5% belonged to extended family households.

General knowledge of participants 

**Figure 1:**
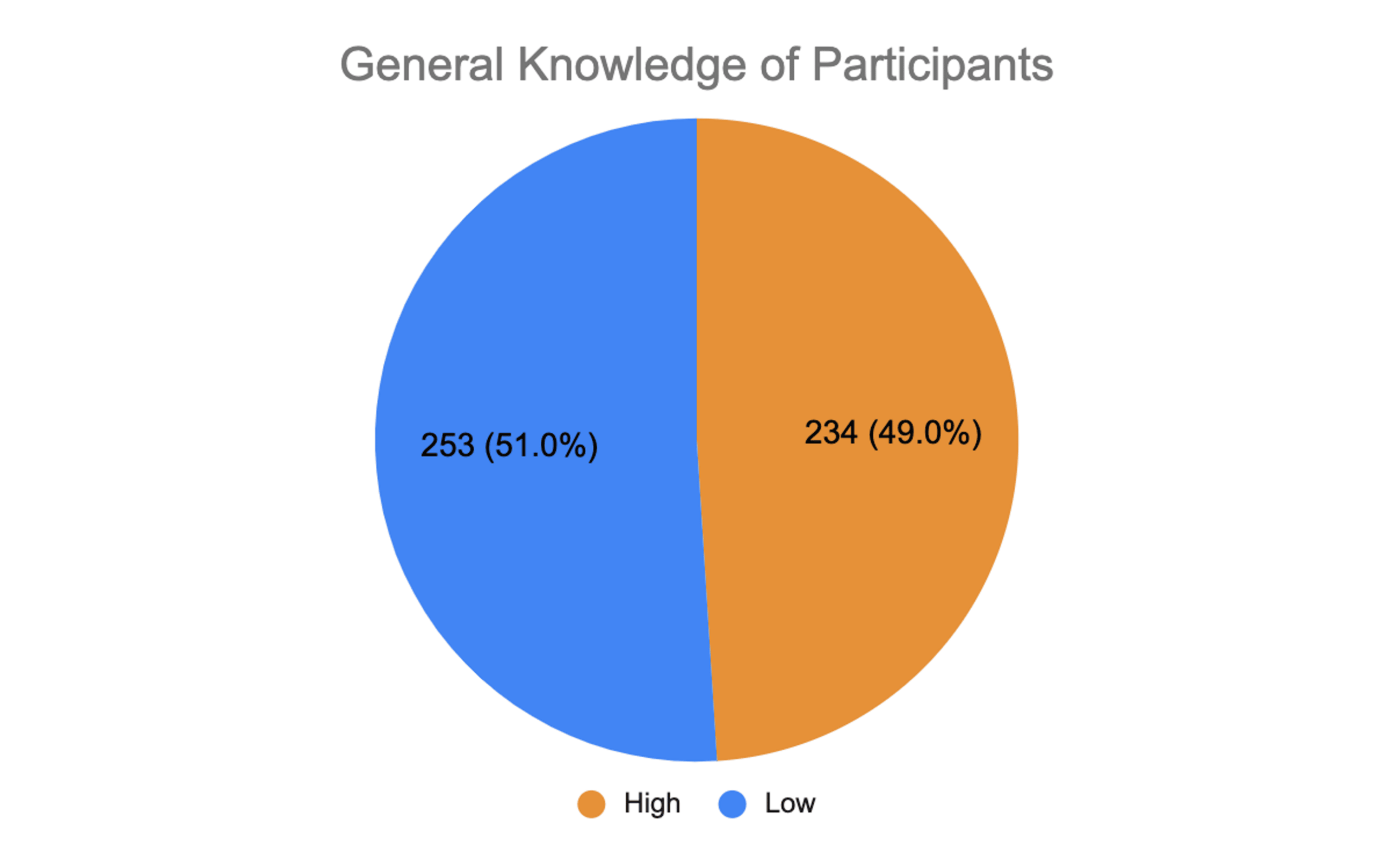
General knowledge of the participants (N = 487)

The figure below depicts the level of general knowledge regarding family planning recorded in the participants. Of note, 51% of the participants demonstrated a low level of knowledge (Figure 1).

Regarding general knowledge, the study identified significant factors (p < 0.001) associated with it, including age, nationality, education, occupation, duration of marriage, number of children, birth spacing, family income, and family type. The overwhelming majority (94.1%) of participants aged 30 years or younger demonstrated a high level of general knowledge about FP methods, which was considerably higher than that of participants in the 31-50 years age group (45.7%) and those over 50 years old (18.3%). 

A high level of knowledge was observed in more than half of Southeast Asian participants (57.5%), compared to 25.5% of participants from the Eastern Mediterranean region. Participants with a university degree or higher (50.6%), those working in the medical field (92.5%), married for 15 years or more (80.8%), participants with no children (96.1%), and those earning AED 15,000 to 25,000 per month (92.9%) also exhibited high levels of knowledge. Conversely, 77.6% of participants with an income exceeding AED 25,000 demonstrated lower knowledge levels. Of note, 51.3% of participants in nuclear families reported high knowledge, compared to 24.2% in extended family households (Table 1).

**Table 1:** Association between general knowledge of family planning methods and sociodemographic variables (N = 487) P-value < 0.05 was statistically significant; the Chi-square test was used AED: United Arab Emirates Dirham

Variable	Subcategories	General Knowledge				Chi-square (χ²) value	P-value
		Low/below average		High			
		Number	%	Number	%		
Gender	Male	134	51.0	129	49.0	2.146	0.143
	Female	129	57.6	95	42.4		
Age, years	≤30	4	5.9	64	94.1	99.016	<0.001
	31-50	165	54.3	139	45.7		
	>50	94	81.7	21	18.3		
Nationality	Southeast Asian	124	42.5	168	57.5	41.418	<0.001
	Eastern Mediterranean	108	74.5	37	25.5		
	Other	31	62.0	19	38.0		
Education	High school and below	52	86.7	8	13.3	29.390	<0.001
	University and above	211	49.4	216	50.6		
Occupation	Medical field	3	7.5	37	92.5	59.364	<0.001
	Non-medical field	205	53.7	177	46.3		
	Unemployed	55	84.6	10	15.4		
Duration of marriage, years	<5	115	38.6	183	61.4	117.060	<0.001
	5-9	71	83.5	14	16.5		
	10-14	72	92.3	6	7.7		
	≥15	5	19.2	21	80.8		
No. of children	0	5	3.9	122	96.1	177.200	<0.001
	1-2	184	68.9	83	31.1		
	3-4	54	77.1	16	22.9		
	≥5	20	87.0	3	13.0		
Birth spacing between the last two children, years	≤2	222	89.5	26	10.5	25.049	<0.001
	≥3	33	62.3	20	37.7		
Monthly family income, AED	<5000	60	59.4	41	40.6	138.663	<0.001
	5000-<15,000	74	62.7	44	37.3		
	15,000-25,000	8	7.1	104	92.9		
	>25,000	121	77.6	35	22.4		
Type of family	Nuclear	191	48.7	201	51.3	22.550	<0.001
	Extended	72	75.8	23	24.2		

Attitude towards family planning 

**Figure 2:**
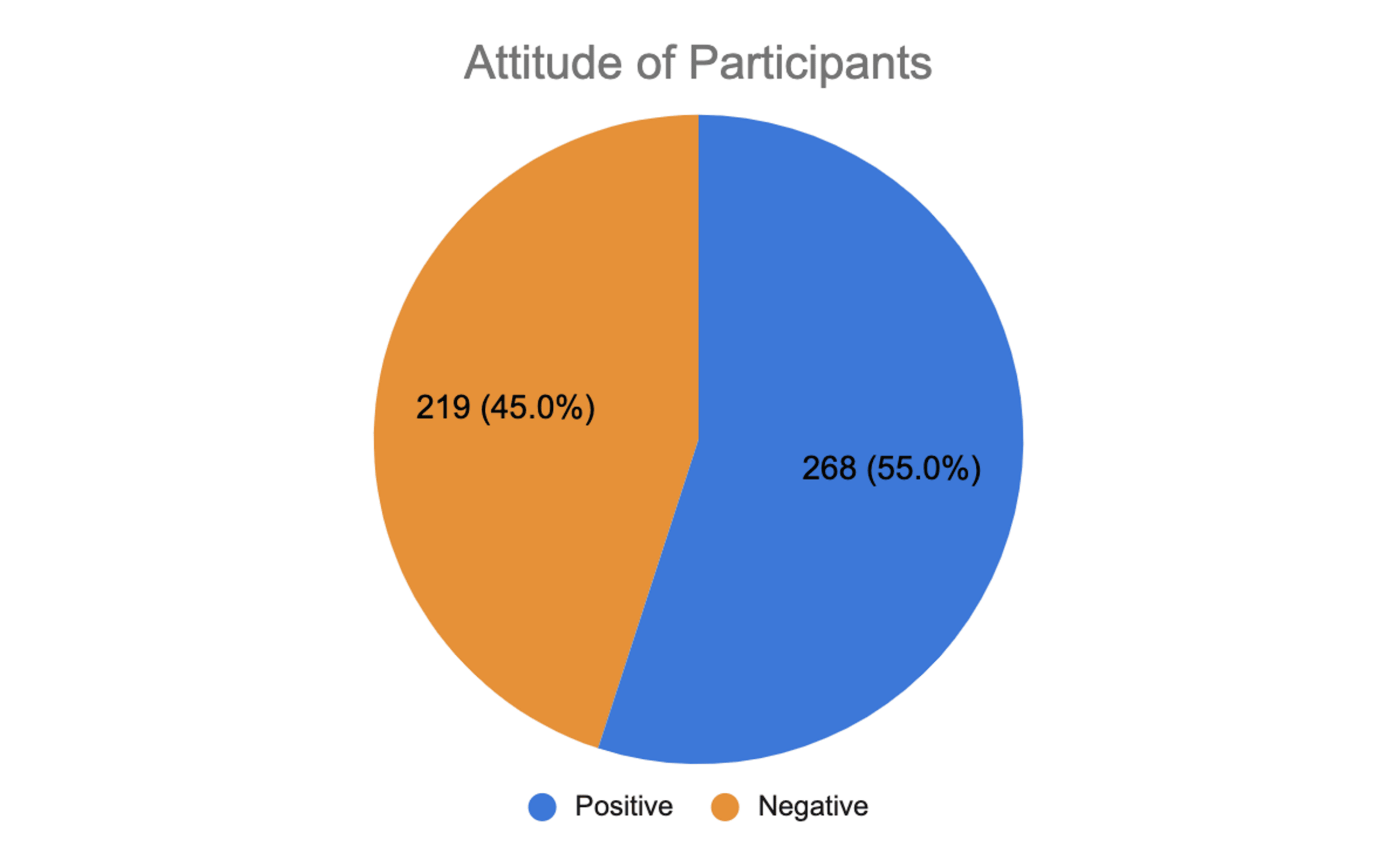
Attitude of participants toward family planning (N = 487)

Figure 2 illustrates the participants’ attitudes toward family planning. Notably, 55% of participants expressed a positive attitude.

Almost all participants (99.2%) reported discussing FP with their spouse, reflecting a positive attitude. Significant factors (p < 0.001) associated with a positive attitude included male gender, Southeast Asian nationality, a university degree or higher, employment, marriage lasting five to nine years, having no children, and living in a nuclear family household (Table 2). 

**Table 2:** Association between attitude towards family planning and sociodemographic factors (N = 487) P-value < 0.05 was statistically significant; the Chi-square test was used AED: United Arab Emirates Dirham

Variable	Subcategories	Attitude towards FP				Chi-square (χ²) value	P-value
		Negative attitude		Positive attitude			
		Number	%	Number	%		
Gender	Male	114	43.3	149	56.7	22.221	<0.001
	Female	145	64.7	79	35.3		
Age group, years	≤30	41	60.3	27	39.7	3.853	0.146
	31-50	165	54.3	139	45.7		
	>50	53	46.1	62	53.9		
Nationality	Southeast Asian	127	43.5	165	56.5	28.374	<0.001
	Eastern Mediterranean	101	69.7	44	30.3		
	Other	31	62	19	38		
Education	High school and below	51	85	9	15	27.823	<0.001
	University and above	208	48.7	219	51.3		
Occupation	Employed	206	48.8	216	51.2	24.223	<0.001
	Unemployed	53	81.5	12	18.5		
Duration of marriage, years	<5	154	51.7	144	48.3	15.711	<0.001
	5-9	34	40	51	60		
	≥10	71	68.3	33	31.7		
No. of children	0	44	34.6	83	65.4	39.483	<0.001
	1-2	143	53.6	124	46.4		
	≥3	72	77.4	21	22.3		
Birth spacing between the last two children, years	≤2	184	74.2	64	25.8	5.276	0.022
	≥3	31	58.5	22	41.5		
Monthly family income, AED	≤15,000	104	48.8	109	51.2	6.026	0.049
	15,000-25,000	81	51.9	75	48.1		
	>25,000	74	62.7	44	37.3		
Type of family	Nuclear	187	47.7	205	52.3	24.225	<0.001
	Extended	72	75.8	23	24.2		

**Figure 3:**
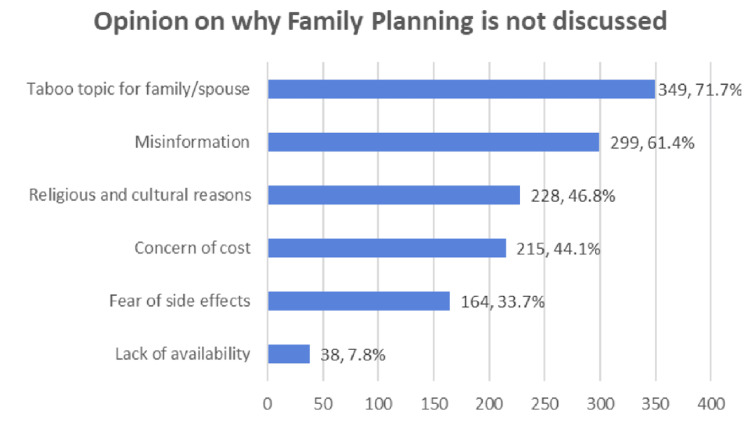
Factors for refusal to accept or discuss FP and contraceptive methods by participants (N = 487) FP: family planning

Participants were asked to indicate their views on the barriers to family planning in the general population. The most frequently reported factors were: taboo topics within the family or with a spouse (71.7%), misinformation (61.4%), religious and cultural reasons (46.8%), concern about cost (44.1%), fear of side effects (33.7%), and limited availability (7.8%) (Figure 3).

Source of knowledge regarding FP methods 

The internet and media (n = 286, 58.7%) was the major source of knowledge regarding family planning among the study population, followed by awareness campaigns (n = 147, 30.2%), education (n = 119, 25.8%), relatives and friends (n = 112, 23%), spouse (n = 90, 18.5%), and healthcare providers (n = 87, 17.9%).

## Discussion

Our study evaluated married men’s and women’s knowledge and attitudes toward family planning methods in the UAE, with approximately half (51%) of participants demonstrating low or below-average knowledge. This underscores existing knowledge gaps in the study population. However, a higher proportion (55%) of participants exhibited a positive attitude and expressed interest in learning more about family planning methods. The percentage of participants with adequate knowledge in this study was lower than that reported in research from Ethiopia and Saudi Arabia, where awareness of family planning methods reached 94% and 80.6%, respectively [4,8]. Similarly, Birabwa et al. interviewed 626 women of childbearing age in Uganda and found that nearly all participants (99.7%) were aware of contraceptive methods [11].

However, findings from other regional studies show considerable variation. For example, a study among women of childbearing age in Makkah, Saudi Arabia, reported that only 44.3% recognized contraception as a method of family planning [12], while a study from Abu Dhabi, UAE, found that just 23.7% of participants demonstrated good knowledge of contraceptive methods [10]. Similarly, research from Northwest Ethiopia reported adequate knowledge in 42.3% of women [13]. These differences likely reflect variations in study populations, measurement tools, definitions of “adequate knowledge,” and sociocultural contexts. When examining associated factors, this study found no statistically significant difference by gender (p = 0.143), although females had a slightly higher proportion of low knowledge compared with males (57.6% vs. 51.0%). This observation aligns with the study by Tilahun et al. [4], which reported comparable median knowledge between genders.

Participant age was strongly associated with knowledge level (p < 0.001). Those aged ≤30 years had the highest proportion of high knowledge (94.1%), whereas participants aged >50 years mostly exhibited low knowledge (81.7%), suggesting that knowledge declines with increasing age. This trend may be explained by greater access to modern sources of information, such as social media. These findings are consistent with a cross-sectional study of 785 women aged 15-49 years in a western district of Odisha, India, which reported significant associations between age and knowledge [14]. Additionally, educational attainment was strongly linked to knowledge, with participants holding higher education degrees demonstrating greater knowledge (50.6%) compared to those with a high school education or below (13.3%). Similar patterns have been reported in studies from Tanzania and India [2,14].

Occupation was another significant factor associated with knowledge. Individuals working in the medical field demonstrated the highest proportion of high knowledge (92.5%), whereas unemployed participants had the lowest proportion (15.4%). Non-medical workers exhibited intermediate levels of knowledge (46.3%). The observed association between occupation and contraceptive knowledge aligns with previous research showing that occupational status affects access to health information [14]. Limited access to reliable family planning information has also been reported as a factor influencing family planning literacy among women in Tanzania [2]. Additionally, a study among married couples in Eastern Ethiopia identified occupation as a determinant of participants’ knowledge of contraceptive methods [15].

Marriage-related factors were also identified as significant determinants of knowledge. Knowledge tended to decrease with longer duration of marriage (p < 0.001), with participants married for 10-14 years showing particularly high proportions of low knowledge (92.3%). In contrast, those married for less than five years demonstrated higher knowledge levels (61.4%). These findings are supported by a large cross-sectional study among married women, which reported that years of marriage significantly affected family planning knowledge and practice, highlighting marital duration as an important sociodemographic factor in reproductive health awareness [16].

Similarly, regarding family characteristics, participants with no children exhibited the highest level of knowledge (96.1%) compared to those with multiple children. The number of children was inversely associated with knowledge (p < 0.001), with only 13.0% of participants having five or more children demonstrating high knowledge levels. This finding aligns with a community-based study in Nepal, which reported that women with two or fewer children were three times more likely to have adequate knowledge of family planning methods, indicating a significant association between the number of children and knowledge [17].

Birth spacing was significantly associated with participants’ knowledge (p < 0.001). Shorter spacing (≤2 years) was linked to notably lower knowledge levels (89.5%), whereas longer spacing (≥3 years) corresponded with relatively higher knowledge. This finding is supported by a study by Yadav et al. in Ethiopia, which reported that knowledge of optimal birth spacing significantly influenced suboptimal birth spacing practices; participants with inadequate knowledge had 2.4 times higher odds of practicing suboptimal birth spacing compared to those with adequate knowledge [18].

Household income demonstrated a complex association with knowledge (p < 0.001). Participants in the middle-income group (AED 15,000-25,000) had the highest proportion of high knowledge (92.9%), whereas both lower-income groups and the highest-income group (>AED 25,000) mostly exhibited low knowledge. This pattern aligns with previous research; a study conducted in the United Arab Emirates reported that family income was significantly associated with women’s contraceptive knowledge, likely reflecting differences in access to education, healthcare services, and information resources [10].

The observed association between family structure and knowledge of family planning methods in this study aligns with previous research, which indicates that women living in nuclear families generally have higher contraceptive knowledge than those in extended or joint family systems, potentially due to greater autonomy, privacy, and access to information [19]. Similarly, a study among married women in Pakistan reported that the type of family system (nuclear vs. joint) significantly influenced both awareness and use of contraceptive methods [20].

Over half of the study population (55%) exhibited a positive attitude toward family planning. This is comparable to studies conducted in Sudan, Saudi Arabia, and Pakistan, where participants also demonstrated positive attitudes despite varying knowledge levels [5,7,9]. However, those studies reported higher proportions of positive attitudes than the current study: 72.5%, 79.6%, and 75.7%, respectively. A cross-sectional study from Fiji reported a similar proportion of positive attitude (56.7%) among pregnant women [21], and a regional study from Abu Dhabi found that 53.5% of participants held a positive attitude [10].

Almost all participants (99.2%) reported discussing family planning with their spouse. This contrasts with findings from Tanzania, where many women had never had such discussions [6], and a cross-sectional household survey from northwestern Nigeria, which found that discussions about fertility and contraceptive methods were uncommon; only 22.5% had ever discussed contraception with their husbands. Despite this, 70% of women agreed that couple discussions about contraception are important. Women who had discussed family planning with their husbands were 14.7 percentage points more likely to be currently using contraceptive methods (21.2% versus 6.5%), highlighting the significant influence of the husband on the intention to initiate contraception [22].

The high proportion of participants in our study reporting discussions with their spouse may reflect cultural expectations or social desirability bias in self-reported responses. Formal education was significantly associated with participants’ attitudes (p < 0.001). Similar findings were reported in a study from the Jimma Zone in Ethiopia, which showed that formal education was linked to more positive attitudes toward family planning [4]. The importance of education was also highlighted in a study conducted in Ankara, Turkey, where women who had completed elementary school or higher demonstrated more positive attitudes, underscoring the role of educational access in shaping women’s perspectives on family planning [23].

Gender was also identified as a significant factor associated with attitude (p < 0.001). Male participants (56.7%) demonstrated a higher proportion of positive attitudes compared with female participants (35.3%). This finding contrasts with a study from India [24], which reported that men’s attitudes were negatively associated with women’s use of family planning methods. In that study, a one standard deviation increase in the proportion of men who believed that contraception is solely the responsibility of women was associated with a 12% decrease in the likelihood of contraceptive use. Similarly, a study from Niger reported negative attitudes among men, particularly regarding women’s involvement in decision-making. Only 5% of men agreed that women should have a role in health-related decisions concerning themselves or their children, while 68.1% believed that such decisions should be made exclusively by men [25].

These findings differ from those of the current study, where almost all participants (99.2%) reported communicating with their spouse about family planning. This underscores the role of perceived gender roles in shaping attitudes toward family planning methods. The higher level of positive attitude observed among men may be influenced by cultural norms, where men may perceive themselves as responsible for implementing family planning decisions to achieve their desired family size. Further investigation of sociodemographic factors linked to male gender, including financial and educational characteristics, may help clarify the reasons behind the higher positive attitudes observed among men.

The significant association between a positive attitude and living in a nuclear household observed in this study aligns with findings from research by Hao et al., which showed that couples in nuclear households had significantly lower fertility intentions compared with those living with parents or in-laws [26]. The same study also reported that belonging to a rural household or an ethnic minority group was associated with significantly higher fertility intentions. Similarly, research conducted across different regions of Pakistan found that participants residing in urban areas were more likely to use contraceptives [27]. These findings emphasize the influence of urbanization and cultural expectations on family planning decisions.

The primary source of information in this study was the internet and media (58.7%), followed by awareness campaigns (30.2%). Similarly, media was identified as a major source of knowledge (35.5%) in a study conducted in Sudan [5], and a study from Saudi Arabia reported that 37.5% of participants obtained their information from the internet. However, in the Saudi study, family members played a larger role as a source of information (51.8%) [8]. In contrast, a study from Quetta reported lower reliance on media, where 28.8% of participants received information from television or radio, while the majority (31.5%) obtained knowledge from family planning services [9].

Despite major advances in modern contraceptive methods, several barriers continue to limit their use. In the present study, the perceived barriers to accepting or discussing family planning included taboo topics within the family or with a spouse (71.7%), misinformation (61.4%), religious and cultural reasons (46.8%), concerns about cost (44.1%), fear of side effects (33.7%), and limited availability (7.8%). These barriers are widely documented in the literature. For example, focus group discussions conducted in Kinshasa identified fear of side effects, the cost of contraceptive methods, sociocultural norms, family influence against modern contraceptive use, and lack of knowledge as key barriers [28]. Similarly, a regional study reported fear of side effects (51.9%) as the main reason for non-use, although this proportion was higher than that observed in the current study [10]. In contrast, a study from Saudi Arabia found that only 11.8% of participants reported a negative attitude due to fear of side effects [8].

Discrepancies in barriers may be related to variations in socioeconomic status and levels of development. A descriptive analysis of 47 countries found that nonuse of contraceptive methods due to “lack of access” or “lack of knowledge” was approximately twice as common in rural areas compared with urban areas. The study also identified pro-rich inequalities in reasons such as “health concerns,” “infrequent sex,” and “method-related factors,” whereas pro-poor inequalities were linked to reasons including “opposition from others,” “fatalistic beliefs,” “lack of access,” and “lack of knowledge” [29]. These findings highlight the importance of understanding demographic and socioeconomic factors to effectively address contraceptive nonuse in both developed and developing regions.

This study has several strengths. The adequate sample size enhances the reliability and reproducibility of the findings. Including both married men and women enabled a more comprehensive assessment of knowledge and attitudes toward family planning. Data collection across multiple healthcare facilities may have contributed to a more diverse study population. Furthermore, the use of a structured and content-validated questionnaire, developed with expert review and pilot testing and covering relevant domains, strengthens the methodological rigor of the study.

Limitations

This study also has several limitations that should be acknowledged. First, the use of a convenience sampling strategy limits the representativeness of the sample and the generalizability of the findings to the broader population. There is a potential for selection bias, as individuals who were accessible and willing to participate may not accurately reflect the larger population. Participants attending healthcare facilities may also have higher educational levels and greater health awareness. Therefore, the findings should be interpreted with caution, as the sample was drawn from individuals visiting Thumbay healthcare facilities and may not fully represent the wider population of the UAE.

Second, the cross-sectional design of the study limits the ability to determine causal relationships. Consequently, only associations can be identified between sociodemographic factors and participants’ knowledge or attitudes toward family planning. Third, the use of an online self-administered questionnaire introduces the possibility of recall bias. Self-reported knowledge may therefore be subject to inaccuracies, as participants might provide responses they perceive as socially desirable. Furthermore, the study included only married individuals due to prevailing social and cultural considerations.

As for the scale applied in the study tool, formal psychometric validation and reliability testing, such as Cronbach’s alpha, were not conducted. Another limitation is that the statistical analysis primarily relied on univariate methods. While the study assessed knowledge and attitudes, it did not evaluate actual contraceptive practices among participants. Future research should aim for larger sample sizes across multiple medical facilities, utilize multivariate regression models to account for potential confounding variables, and implement random sampling methods.

## Conclusions

Our study concluded that although the level of knowledge among the majority of participants was low or below average (51%), over half of the participants demonstrated a positive attitude (55%) and expressed an interest in learning more about FP. Factors significantly associated with the level of knowledge included sociodemographic and family characteristics, such as age, education, occupation, income, duration of marriage, number of children, and household structure. Factors associated with a positive attitude were being male, of Southeast Asian nationality, having a university education or higher, being employed, married for five to nine years, having no children, and living in a nuclear family household. The findings highlight potential opportunities to implement awareness campaigns and educational programs aimed at improving understanding of the topic. Counseling sessions that emphasize the importance of FP and address misconceptions could be beneficial for the general population. Further research investigating contraceptive practices and behavioral outcomes would help determine the impact of such initiatives.

## Appendices

Appendix 1: study questionnaire

**Table 3:** Study questionnaire FP: family planning

Section	Items	Response categories
Sociodemographic data	Gender	Male/female
	Age	Open response
	Nationality	Open response
	Highest education level	≤Primary school
		High school/secondary school
		University or above
	Occupation	Medical field
		Non-medical field
		Unemployed
		Retired
	Duration of marriage (years)	<5
		5-9
		10-14
		≥15
	Number of children	0
		1-2
		3-4
		≥5
	If you have more than one child, please select the option that shows the space (in years) between the last two children	<1
		1-2
		3-4
		≥5
	Total family income per month	Less than 5,000 AED
		5,000-15,000 AED
		15,000-25,000 AED
		More than 25,000 AED
	Type of family structure	Nuclear (no parent/relatives of either husband or wife)
		Extended (live with parents/relatives of either husband or wife)
General knowledge domains on FP	Goal of FP	Very high/high/average/low/very low/never heard of
	Effectiveness of different FP methods	Very high/high/average/low/very low/never heard of
	Side effects of different FP methods	Very high/high/average/low/very low/never heard of
	How to use different FP methods	Very high/high/average/low/very low/never heard of
	What are the precautions to be considered when using some FP methods	Very high/high/average/low/very low/never heard of
	Effect of FP methods on fertility and risk of sexually transmitted diseases/genital infection	Very high/high/average/low/very low/never heard of
Source of knowledge	What is the source of your knowledge about FP?	Relatives and friends
		Spouse
		Internet and media
		Awareness campaigns
		Healthcare providers, such as a doctor or nurse
		Other:
		None
Additional	Do you communicate with your spouse about FP:	Yes/no
Attitude towards FP methods	Partner discussion about FP is important	Strongly agree/agree/neutral/disagree/strongly disagree
	Do you have an interest in learning about FP	Strongly agree/agree/neutral/disagree/strongly disagree
	Spreading awareness of FP is important	Strongly agree/agree/neutral/disagree/strongly disagree
	Using FP is important for women	Strongly agree/agree/neutral/disagree/strongly disagree
	Using FP is important for men	Strongly agree/agree/neutral/disagree/strongly disagree
	Large family size affects the development of a family	Strongly agree/agree/neutral/disagree/strongly disagree
	Advising other women for FP is good\appropriate	Strongly agree/agree/neutral/disagree/strongly disagree
	The use of oral contraceptive pills is inconvenient	Strongly agree/agree/neutral/disagree/strongly disagree
	Contraceptive information should only be given to married couples	Strongly agree/agree/neutral/disagree/strongly disagree
	FP is not a restricted topic in my community	Strongly agree/agree/neutral/disagree/strongly disagree
	I believe in the effectiveness of contraceptive methods	Strongly agree/agree/neutral/disagree/strongly disagree
	I believe oral contraceptives are safe	Strongly agree/agree/neutral/disagree/strongly disagree
	I would use oral contraceptives/let my partner use oral contraceptives	Strongly agree/agree/neutral/disagree/strongly disagree
	I want to get a vasectomy/tubal ligation in the future	Strongly agree/agree/neutral/disagree/strongly disagree
	Married couples are shy to talk about contraception with each other	Strongly agree/agree/neutral/disagree/strongly disagree
	In your opinion, why might people rarely discuss or refuse to accept FP and contraceptive methods (select all that apply)	Fear of side effects
		Concern about cost
		Misinformation
		Taboo topic for family/spouse
		Religious and cultural reasons
		Lack of availability
		Other: ________
